# Residential Exposure to Traffic-Related Air Pollution and Survival after Heart Failure

**DOI:** 10.1289/ehp.10918

**Published:** 2008-01-10

**Authors:** Mercedes Medina-Ramón, Robert Goldberg, Steven Melly, Murray A. Mittleman, Joel Schwartz

**Affiliations:** 1 Department of Environmental Health, Harvard School of Public Health, Boston, Massachusetts, USA; 2 Department of Community Health, Brown University, Providence, Rhode Island, USA; 3 Department of Medicine, University of Massachusetts Medical School, Worcester, Massachusetts, USA; 4 Department of Epidemiology, Harvard School of Public Health, Boston, Massachusetts, USA; 5 Cardiovascular Epidemiology Research Unit, Beth Israel Deaconess Medical Center, Boston, Massachusetts, USA

**Keywords:** air pollution, epidemiology, follow-up studies, heart failure, survival

## Abstract

**Background:**

Although patients with heart failure (HF) have been identified as particularly susceptible to the acute effects of air pollution, the effects of long-term exposure to air pollution on patients with this increasingly prevalent disease are largely unknown.

**Objective:**

This study was designed to examine the mortality risk associated with residential exposure to traffic-related air pollution among HF patients.

**Methods:**

A total of 1,389 patients hospitalized with acute HF in greater Worcester, Massachusetts, during 2000 were followed for survival through December 2005. We used daily traffic within 100 and 300 m of residence as well as the distance from residence to major roadways and to bus routes as proxies for residential exposure to traffic-related air pollution. We assessed mortality risks for each exposure variable using Cox proportional hazards models adjusted for prognostic factors.

**Results:**

After the 5-year follow-up, only 334 (24%) subjects were still alive. An interquartile range increase in daily traffic within 100 m of home was associated with a mortality hazard ratio (HR) of 1.15 [95% confidence interval (CI), 1.05–1.25], whereas for traffic within 300 m this association was 1.09 (95% CI, 1.01–1.19). The mortality risk decreased with increasing distance to bus routes (HR = 0.88; 95% CI, 0.81–0.96) and was larger for those living within 100 m of a major roadway or 50 m of a bus route (HR = 1.30; 95% CI, 1.13–1.49). Adjustment for area-based income and educational level slightly attenuated these associations.

**Conclusions:**

Residential exposure to traffic-related air pollution increases the mortality risk after hospitalization with acute HF. Reducing exposure to traffic-related emissions may improve the long-term prognosis of HF patients.

Long-term exposure to particulate air pollution has been associated with increases in mortality, especially in cardiopulmonary mortality (Dockery et al. 1993; Hoek et al. 2002; Jerrett et al. 2005; Miller et al. 2007; Pope et al. 2002). Even though the specific components responsible for this association remain unknown, there is some evidence indicating that traffic-related air pollution may be particularly harmful (Finkelstein et al. 2004; Hoek et al. 2002; Tonne et al. 2007).

Assessment of the effects of air pollution on potentially susceptible subpopulations is key to providing policy-relevant information to better protect the vulnerable. Studies of the acute effects of air pollution have identified patients with heart failure as being particularly susceptible to the effects of particulate air pollution (Goldberg et al. 2001; Hoek et al. 2001a; Schwartz and Morris 1995; Wellenius et al. 2005); however, little is known about the long-term effects of air pollution on patients with this prevalent and highly fatal clinical syndrome.

Using data from an ongoing population-based investigation of the incidence and prognosis of heart failure (Goldberg et al. 2005, 2007), we examined the mortality risk associated with residential exposure to traffic-related air pollution among patients hospitalized with acute heart failure.

## Materials and Methods

### Study design and population

The present analysis was conducted as part of the Worcester Heart Failure Study, an ongoing investigation describing the epidemiology of heart failure in residents of the greater Worcester, Massachusetts, area (Goldberg et al. 2005, 2007). The study was approved by the Committee for the Protection of Human Subjects in Research from the University of Massachusetts Medical School and the Human Subjects Committee from the Harvard School of Public Health. Our study sample included all adult residents of greater Worcester who had been hospitalized with heart failure and discharged alive during 2000. We reviewed the hospital records from all 11 medical centers in the Worcester Metropolitan Statistical Area to identify subjects with primary or secondary discharge diagnoses consistent with the possible presence of heart failure. We selected for review the following diagnostics: heart failure [*International Classification of Diseases, Ninth Revision*, (ICD-9; World Health Organization 1975): code 428], rheumatic heart failure (ICD-9: 398.91), hypertensive heart disease (ICD-9: 402), hypertensive heart and kidney disease (ICD-9: 404), acute cor pulmonale (ICD-9: 415.0), pulmonary heart disease (ICD-9: 416.9), other diseases of endocardium (ICD-9: 424), primary cardiomyopathies (ICD-9: 425.1, 425.4, and 425.5), pulmonary congestion (ICD-9: 514), acute lung edema (ICD-9: 518.4), edema (ICD-9: 782.3), dyspnea and respiratory abnormalities (ICD-9: 786.0), and chest pain (ICD-9: 786.5). After review of the medical records, those who fulfilled the Framingham criteria for congestive heart failure (McKee et al. 1971; Roger et al. 2004) and were residents of the Worcester metropolitan area were included in our cohort. Individual addresses were obtained for all these subjects. For patients hospitalized more than once during 2000, data from the first hospitalization was used (here referred to as index hospitalization). Vital status at the end of follow-up (December 2005) was ascertained using the U.S. Social Security Death Index (http://www.ntis.gov/products/ssa-dmf.aspx) as well as data from the Massachusetts Registry of Vital Records and Statistics (http://www.mass.gov/?pageID=eohhs2terminal&L=5&L0=Home&L1=Government&L2=Departments+and+Divisions&L3=Department+of+Public+Health&L4=Programs+and+Services+K+-+S&sid=Eeohhs2&b=terminalcontent&f=dph_vital_records_g_about&csid=Eeohhs2).

### Data collection

Information obtained from hospital medical records included demographic characteristics, medical history, and the results of physiologic measures and laboratory tests performed during hospitalization. The medical records of previous hospitalizations or outpatient visits for heart failure were also reviewed to ascertain a prior history of heart failure, as well as other comorbidities in the study sample.

### Exposure assessment

We used ArcGIS software (version 9; ESRI, Redlands, WA) to assign exposure to traffic-related air pollution to each patient based on their geocoded residence at the time of the index hospitalization. A precision of at least a nine-digit ZIP code (usually corresponding to a single side of a city block in urban areas or to both sides of a longer roadway in rural areas) was required for inclusion in the study. We used three different measures as surrogates for exposure to traffic-related air pollution: daily traffic near the patient’s residence, distance to a major roadway, and distance to a bus route.

Using data from the *2002 Road Inventory* from the Massachusetts Highway Department (2002), we calculated the daily traffic within 100 m and 300 m of the patient’s residence. For each road, we calculated its traffic contribution as the product of the length of the road segment falling within the circle and its annual average daily traffic (i.e., the average number of vehicles circulating on the road each day). The traffic contributions from each road were then added to obtain the overall daily traffic within the circle area. We also calculated the distance from the patient’s residence to the nearest primary highway with limited access (U.S. Census Bureau’s feature class A1) using StreetMap 8.3 (ESRI). Finally, we calculated the distance to the nearest public bus route using data from the Central Massachusetts Regional Planning Commission (Worcester, MA; Morin M, personal communication).

As a surrogate for socioeconomic status (SES), we used area-based measures of income and educational attainment available from the 2000 U.S. Census (U.S. Census Bureau 2000). Both the median household income in 1999 and the percentage of persons ≥ 25 years of age whose highest degree was a high school diploma were determined at the census block group level and then assigned to each participant according to the location of residence. In the United States, a block group typically contains about 1,500 people, and is designed to be relatively homogeneous on demographic characteristics (U.S. Census Bureau 2000, 2001).

### Data analysis

First, we assessed the association between residential exposure to traffic and survival after acute heart failure by constructing unadjusted Kaplan–Meier survival curves for the low and high exposure groups of each exposure variable. For variables measuring the daily traffic near the patient’s residence, the low exposure group was defined as those with an exposure less than that of the 25th percentile and the high exposure group as those with an exposure greater than that of the 75th percentile. Conversely, for variables measuring distance to traffic arteries, the low and high exposure groups were defined using as cutoff points the respective 75th and 25th percentiles of the variable’s distribution.

Subsequently, we used Cox proportional hazards regression models to assess the association between survival after acute heart failure and traffic in a continuous scale, controlling for other potential risk factors. Analyses were performed using the PROC PHREG procedure in SAS software (version 9.1; SAS Institute Inc., Cary, NC). The baseline survival model included as explanatory variables all variables presented in Table 1, which were selected *a priori* based on a previous analysis looking at survival after heart failure within the same cohort (Goldberg et al. 2007). Because the proportional hazards assumption was violated by the variables “systolic blood pressure” and “previous heart failure,” we included an interaction term between “systolic blood pressure” and the logarithm of survival time, and used “previous heart failure” as a stratification variable in the model. Continuous variables used as surrogates of traffic-related exposure were logarithmically transformed and introduced, one at a time, in the baseline model. Here we present the mortality risks associated with an interquartile range (IQR) change in exposure (i.e., the risk associated to increasing the exposure from the 25th percentile to the 75th percentile of the distribution of each exposure variable).

As a sensitivity analysis, we repeated the analyses adjusting the baseline model first for household income level and then for educational attainment at the block group level. For a subset of individuals with complete smoking information (84%), we repeated the baseline analysis additionally adjusting for smoking status.

## Results

A total of 1,752 greater Worcester residents were discharged alive after a confirmed episode of acute heart failure during 2000. Of these, 1,642 (94%) had a home address that could be accurately geocoded (1,384 to the exact street address). After excluding individuals without complete information on the demographic and clinical characteristics used in our baseline analysis, the final sample size was 1,389 patients. The mean age of the study population was 76 years, slightly more than half (56%) were women, and most (92%) were white (Table 1). Approximately two-thirds of the patients had a prior history of heart failure and a medical history of other cardiovascular diseases. The median time of survival was 2.04 years, and by the end of the 5-year follow-up, only 334 (24%) subjects were still alive. The median household income at the block group level ranged from $9,561 to $177,361 (mean = $55,231) and the percentage of low-educated persons from 12% to 93% (mean = 50%). Data on smoking status were available for 1,164 patients (84% of the final study sample). Of these, 52% were ex-smokers and 12% current smokers at the time of hospitalization.

Table 2 shows the distribution of the exposure variables in the study population. The median daily traffic within 300 m of the patient’s residence (4,541 vehicle-km) was about 10-fold greater than the median daily traffic within 100 m (448 vehicle-km). Overall, the distance to a nearby bus route (median = 226 m) was smaller than the distance to a major roadway (1,640 m). The IQR differences ranged from about a 3-fold increase for distance to a major roadway to 20-fold increase for distance to a bus route. The median daily traffic was lower for those patients who were still alive by the end of follow-up, and the distance between their residence and traffic arteries was larger.

**Figure 1:**
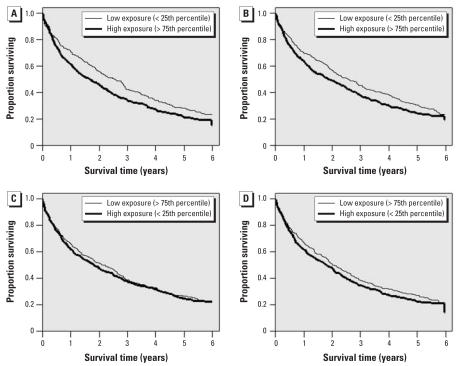
Long-term survival for the low and high exposure groups of each surrogate of traffic-related air pollution exposure. Daily traffic within 100 m (*A*) and 300 m (*B*), and distance to major roadway (*C*) and bus route (*D*).

Crude differences in the survival curves between the low and high exposure groups were statistically significant only for daily traffic within 100 m (log-rank *p*-value: 0.017) (Figure 1). Although less apparent, lower survival rates were also observed for patients with high daily traffic within 300 m of their residence and those living in closer proximity to a bus route.

In the baseline model, after adjustment for all covariates presented in Table 1, the risk of dying associated with an IQR increase in daily traffic within 100 m of the patient’s residence was 1.15 [95% confidence interval (CI), 1.05–1.25] (Table 3). A weaker association, although also statistically significant, was observed for traffic within 300 m of the patient’s home. The risk of dying after hospital discharge decreased with increasing distance to a bus route, with a hazard ratio (HR) of 0.88 (95% CI, 0.81–0.96) for an IQR increase in distance, whereas distance to major roadways did not significantly change the risk. Overall, adjustment for SES reduced the size of effect estimates. However, traffic within 100 m of the patient’s residence (*p* < 0.05) and distance to a bus route (*p* < 0.1) remained as important risk factors for mortality after hospitalization for heart failure. Adjustment of the baseline model for smoking status led to essentially the same results.

## Discussion

The results of our large population-based study among patients with acute heart failure suggest an increased risk of dying associated with exposure to traffic near the patient’s residence. A greater impact on mortality was observed for exposure to traffic at the local scale than for traffic circulating on larger roadways at greater distances, which could be explained partially by a different composition of the pollutants mixture at these two scales. Air pollutants undergo physical and chemical changes after emission, and our results suggest that primary emissions may be more important than the aged emissions.

Consistent with our results, cohort studies in the general population have found an increased risk of dying associated with long-term exposure to particulate air pollution (Dockery et al. 1993; Jerrett et al. 2005; Miller et al. 2007; Pope et al. 2002). Living near a major road has also been associated with total (Finkelstein et al. 2004; Hoek et al. 2002), cardiopulmonary (Gehring et al. 2006; Hoek et al. 2002), and stroke (Maheswaran and Elliott 2003) mortality as well as with a higher prevalence of coronary heart disease (Hoffmann et al. 2006). To our knowledge, no study has examined the long-term effects of traffic-related air pollution in heart failure patients. There is some evidence, however, that patients with acute heart failure may be at an increased risk for dying from the acute effects of air pollution (Goldberg et al. 2001; Kwon et al. 2001). Moreover, increases in heart failure hospital admissions (Barnett et al. 2006; Dominici et al. 2006; Wellenius et al. 2005) and deaths (Hoek et al. 2001a) have been related to short-term elevations in traffic-related air pollutants, such as carbon monoxide, nitrogen dioxide, and fine particulate matter.

Individuals with a high long-term residential exposure to traffic are also more likely to have episodes of high acute exposure to traffic-related air pollution. Thus, even though we used a long-term metric to assign exposure to traffic, the observed effects in our study would probably be the result of both long-term and acute exposure. Comparison of our results with those from case–crossover and time-series studies investigating the acute effects of air pollution in heart failure patients (Goldberg et al. 2001; Kwon et al. 2001) shows that our effect estimates are two to three times larger. This suggests an important contribution of sustained long-term exposure to traffic to the underlying mechanisms related to mortality in heart failure patients.

Because of their condition, their advanced age, and the common presence of other comorbidities, heart failure patients may be particularly susceptible to the long-term effects of traffic-related air pollution. To test this hypothesis by comparing our results with those obtained in other populations, we repeated our analyses using a similar approach to that followed in two other cohort studies in which long-term exposure to traffic was defined as living within 100 m of a highway or within 50 m of a major urban road (in our case, a bus route). In our study, the resulting mortality HR was 1.30 (95% CI, 1.13–1.49), which was greater than that obtained in a 10-year follow-up of Canadian subjects who underwent pulmonary function tests (HR = 1.18; 95% CI, 1.02–1.38) (Finkelstein et al. 2004). In contrast, our estimate was slightly smaller than that obtained in a cohort study among the Dutch general population (HR = 1.41; 95% CI, 0.94–2.12), where background levels of air pollution were taken into account (Hoek et al. 2002). The latter study, however, found larger effect estimates for cardiopulmonary mortality (HR = 1.95; 95% CI, 1.09–3.51) than for all-cause mortality, which is consistent with an increased susceptibility to the effects of air pollution in heart failure patients. Overall, the effects of residential exposure to traffic in our study and the aforementioned studies were of similar magnitude, and differences attributable to varying susceptibility of populations are hard to discern given other differences in methodology and geographic location of theses studies.

In our study, traffic within 100 m of the patient’s residence was a stronger determinant of mortality after heart failure than traffic within 300 m, which in turn was a stronger determinant than distance to major roadway. This suggests that local traffic exposure plays a more relevant role in determining survival after heart failure than traffic circulating at greater distances. In agreement with our findings, the aforementioned Dutch cohort study found that the mortality risk associated with living within 100 m of a major road was higher than that associated with the urban background concentration of traffic-related air pollution (Hoek et al. 2002). Both the concentration of air pollution and its composition vary with distance to the emission source. Concentrations of freshly emitted traffic-related pollutants are much higher near streets/roads and drop off to the local background concentration beyond 100 m (Roorda-Knape et al. 1998). Compared with more aged emissions, fresh traffic emissions include larger amounts of ultrafine particles, which are strongly oxidative and may be particularly toxic (Nel 2005). Elemental carbon concentrations also fall to local background levels within 100 m of roads. Diesel engines are the strongest sources of these two pollutants (Morawska et al. 1998).

Proximity to traffic arteries seemed to play a less important role in determining survival after heart failure, especially after adjustment for SES. However, the results for distance to bus routes were marginally significant (< 0.1), and the association was in the expected direction (i.e., lower mortality risks as distance increased). The substantial difference between the effect estimates and significance for distance to a bus route compared with distance to a major roadway suggests that composition of the traffic pollution may matter. In our study area, bus routes are expected to have more diesel-fueled vehicles (buses but also trucks) than major roadways or local streets. Compared with gasoline engines, diesel engines emit larger amounts of nitrogen oxides, aldehydes, and particles, including sub-micron soot particles (elemental carbon) and ultrafine particles (Sydbom et al. 2001). In a study in a nearby town in greater Boston, Massachusetts, concentrations of fine particulate matter and particle-bound polycyclic aromatic hydrocarbons were higher on bus routes, particularly on streets with elevated bus traffic (Levy et al. 2001). It is also worth noting that because bus routes are usually more transited than other streets, residences located at a smaller distance to a bus route presented also high traffic densities within 100 m (Pearson correlation = – 0.48; *p* < 0.001). Thus, distance to bus routes could also be acting as a surrogate for high traffic density near home.

The adverse effects of traffic-related air pollution on survival after heart failure persisted even after adjustment for area-based SES measures, although the associations were generally attenuated and the effect of distance to a bus route was only marginally significant. Even though census tract–level SES measures have been shown to consistently detect socioeconomic gradients in mortality (Krieger et al. 2002; Tonne et al. 2005), this ecologic measure is less accurate than individual-level SES measures and some residual confounding can be expected. Previous studies have shown that individuals with lower SES have generally poorer health status and live shorter lives (Kaplan and Keil 1993; Marmot and Feeney 1997). This could be related, among other factors, to a limited access to health care and/or effective medications as well as to a lower degree of compliance with treatment among the most socially disadvantaged. However, this could also reflect characteristics of the physical environment, including housing conditions and residential exposure to traffic. Thus, although examining the effect of SES as a confounder in our study is important, estimates derived from the SES-adjusted models should be interpreted as conservative because they may be overadjusted.

One of the several hypothesized biological pathways through which air pollution may increase cardiovascular mortality is by alterations of the cardiac autonomic function (Kwon et al. 2001; Pope et al. 2004; Routledge and Ayres 2005). Recent epidemiologic studies have reported an association between increases in particulate matter and reduced heart rate variability (Gold et al. 2000; Pope et al. 1999), which is an independent predictor of mortality in patients with chronic heart failure (La Rovere et al. 2003; Nolan et al. 1998; Ponikowski et al. 1997). In a recent study among elderly subjects (Schwartz et al. 2005), traffic particles were reported to have a stronger impact on heart rate variability than other particles or gaseous pollutants. Thus, a plausible explanation for our findings would be that traffic-related air pollution may increase mortality in heart failure patients by altering their cardiac autonomic function. Furthermore, several recent studies have documented that acute exposure to traffic-related particulate air pollution is associated with an increased risk of ventricular arrhythmias among patients with implanted cardioverter-defibrillators, many of whom have depressed left ventricular ejection fractions and an element of heart failure (Dockery et al. 2005; Rich et al. 2006).

An important limitation of our study is the potential for misclassification of our key exposure variables. In the first place, wind patterns and topography may alter the spatial distribution of air pollutants, which was not taken into account in our exposure variables. For instance, for a given distance between residence and the nearest bus route, exposure to traffic-related air pollution may be different for those living upwind or downwind. Nevertheless, distance to motorways and traffic density near residence have been shown to be good proxies for actual outdoor concentrations of traffic-related air pollutants in other studies (Brauer et al. 2003; Hochadel et al. 2006; Hoek et al. 2001b; Janssen et al. 2001). In turn, exposure outside the residence is a better surrogate for personal exposure to particles of outdoor origin than central station monitoring. However, outdoor pollution at the residential location is just an approximation of participants’ true exposure. Exposure to pollutants will be influenced not only by the degree of correlation between outdoor and indoor pollution levels, but also by the amount of time that participants spent at home (and other places), infiltration rates, and length of residence in that location. The slope of the personal/outdoor particle relationship has been shown to vary with infiltration rate (Sarnat et al. 2000). In general, when windows are shut and infiltration is low, the slope is only about 60% as large as when infiltration is high, but even then averages over 0.4. For gaseous pollutants from traffic, the associations are weaker than for particles (Sarnat et al. 2005). The lack of information about these factors may have led to non-differential exposure misclassification, which would result in an underestimation of the risk associated with traffic-related air pollution. However, in our study, with elderly infirm subjects, an estimate of concentration outside their home is probably a better surrogate for their exposure than in a more mobile population.

A strength of our study is that the diagnosis of heart failure was independently confirmed using a standardized review of hospital medical records, and that the entire population of subjects hospitalized for heart failure was examined, reducing the likelihood of selection bias. However, this limits the extrapolation of results to individuals with a less severe heart failure, namely, those who had a heart failure during the study enrollment period but were not hospitalized, which in Olmsted County, Minnesota, represented about a quarter of all heart failure incident cases (Roger et al. 2004). In addition, we lost about 21% of patients because of bad address (6%) or missing data in any of the covariates of the baseline model (15%), which somewhat limits the extrapolation of our results to those patients whose medical records are incomplete.

The public health impact of traffic-related air pollution on heart failure patients can be considerable. Even though the effect sizes of our exposure variables were not dramatic, variations within the normal range were associated with mortality increases on the order of 15%, and for those highly exposed this mortality increase was as high as 30%. Given the large mortality rates among heart failure patients (with a median survival time of 2 years) and the sustained increase in heart failure prevalence during the past decades in the United States (Kannel 2000), a 15–30% increase in mortality constitutes an important additional burden.

In conclusion, the results of our study in a large New England community suggest that residential exposure to traffic-related air pollution increases the risk of dying after hospitalization for heart failure. Exposures occurring close to the personal residence, and possibly those related to diesel engines, may be more hazardous. Given the poor long-term prognosis of patients hospitalized with heart failure (Goldberg et al. 2007; Kannel et al. 1994), our findings are of considerable public health concern. Clinicians and policy makers need to consider where these patients live and methods to reduce exposure to traffic-related emissions, to improve the long-term prognosis of patients with this increasingly prevalent clinical syndrome (Kannel 2000; Roger et al. 2004).

## Figures and Tables

**Table 1:** Demographic and clinical characteristics of the study population (*n* = 1,389)

Characteristic	Value
Demographic characteristics	
Age [years (mean ± SD)]	76.1 ± 12.5
Male (%)	43.8
White race (%)	92.3
Medical history (%)	
Heart failure^a^	67.0
Anemia	21.2
Alzheimer disease or dementia	9.4
Atrial fibrillation	34.6
Cardiovascular disease^b^	68.3
Cerebrovascular disease^c^	15.1
Chronic lung disease	32.3
Diabetes	26.4
Liver disease/failure	3.0
Peripheral vascular disease	15.5
Renal disease^d^	21.4
Valvular heart disease^e^	23.4
Symptoms during physical exams (%)	
Dyspnea	93.7
Edema/swelling	71.7
Orthopnea	37.3
Pulmonary rales	91.5
Days of hospitalization (mean ± SD)	5.8 ± 7.4
Vital signs at discharge (mean ± SD)	
Heart rate (beats/min)	75.8 ± 14.0
Systolic blood pressure (mmHg)	128.1 ± 21.4
Blood tests at discharge (mean ± SD)	
Blood urea nitrogen (mg/dL)	35.2 ± 23.0
Creatinine (mg/dL)	1.5 ± 1.2
Glucose (mg/dL)	131.3 ± 57.3
Glomerular filtration rate (mL/min/1.73 m^2^)	64.9 ± 33.2
Hematocrit (%)	35.3 ± 5.7
Sodium (mEq/L)	138.3 ± 19.4
Alive by the end of the 5-year follow-up (%)	24.1

a Hospitalization for heart failure or diagnostic or treatment for heart failure occurring before the index hospitalization.

b Angina pectoris, asystole, ventricular fibrillation, coronary angioplasty, coronary heart disease, left ventricular hypertrophy, and/or myocardial infarction.

c Stroke and/or transient ischemic attack.

d Dialysis and/or renal failure/disease.

e Aortic stenosis, mitral stenosis, mitral regurgitation, aortic insufficiency, and/or tricuspid regurgitation.

**Table 2:** Percentile distribution of traffic exposure variables in the study population.

	All subjects						
Exposure	5%	25%	50%	75%	95%	Dead^a^ (50%)	Alive^a^ (50%)
Daily traffic within 100 m (vehicle-km)	45	200	448	1,579	4,937	462	396
Daily traffic within 300 m (vehicle-km)	434	1,964	4,541	11,389	33,589	4,613	4,366
Distance to major roadway (m)	238	891	1,640	2,899	6,687	1,603	1,700
Distance to bus route (m)	5	43	226	882	6,968	222	226

a Vital status at the end of the 5-year follow-up (December 2005).

**Table 3:** Association between traffic exposure variables and survival after hospital discharge for heart failure.

	Baseline model		Adjusted for income		Adjusted for education	
Exposure	HR^a^ (95% CI)	*p*-Value	HR^a^ (95% CI)	*p*-Value	HR^a^ (95% CI)	*p*-Value
Daily traffic within 100 m (vehicle-km)	1.15 (1.05–1.25)	0.002	1.13 (1.03–1.23)	0.01	1.12 (1.03–1.23)	0.01
Daily traffic within 300 m (vehicle-km)	1.09 (1.01–1.19)	0.03	1.07 (0.98–1.16)	0.16	1.06 (0.98–1.16)	0.16
Distance to major roadway (m)	0.98 (0.91–1.05)	0.56	1.00 (0.93–1.08)	0.94	1.02 (0.94–1.10)	0.69
Distance to bus route (m)	0.88 (0.81–0.96)	0.005	0.91 (0.82–1.00)	0.07	0.91 (0.83–1.01)	0.08

All models are adjusted for variables presented in Table 1.

a Mortality HRs associated with an IQR change in exposure.
